# An Anxious Doctor

**Published:** 1891-07

**Authors:** 


### An Anxious Doctor.

The following taken from *The Medical News*, is a letter received by an Elkhart, Ind., physician from a country doctor (?):

"Dear dock I hav a pashant whos phisicol sines shoes that the wind-pipe has ulcerated of, and his lung have drop intoo his stumick. he is unabel to swoller and I feer his stumic tube is gon. I hav giv hym evrything without efeckt. his father is welthy, Onerable and influensihial. he is an active membber of the M. E. Chirsch and god nose I dont want to loose hym. what shall I due. ans. buy returne male.

Yours in neede."

ETHER drinking among the Poles, Finns and Swedes in the northern counties of the State of Michigan, is reported. They use the compound spirits of ether and it is generally consumed with alcohol or whisky.

ARTIFICIAL quinine is the latest discovery. M. M. Grimaux and Arnaud have realized this chemical dream and report their process as follows: The base cuprein contained in the shrub Remijia pedunculata, growing in Brazil, is treated with sodium then the combination thus obtained is chloride of methyl. The product is quinine absolutely identical with the substance with which we are familiar.

